# Improvement in Isolation and Identification of Mouse Oogonial Stem Cells

**DOI:** 10.1155/2016/2749461

**Published:** 2015-11-09

**Authors:** Zhiyong Lu, Meng Wu, Jinjin Zhang, Jiaqiang Xiong, Jing Cheng, Wei Shen, Aiyue Luo, Li Fang, Shixuan Wang

**Affiliations:** ^1^Department of Obstetrics and Gynecology, Tongji Hospital, Tongji Medical College, Huazhong University of Science and Technology, 1095 Jiefang Road, Wuhan, Hubei 430030, China; ^2^Hubei Key Laboratory of Embryonic Stem Cell Research, Tai-He Hospital, Hubei University of Medicine, Shiyan, Hubei, China

## Abstract

Female germline stem cells (FGSCs) or oogonial stem cells (OSCs) have the capacity to generate newborn oocytes and thus open a new door to fight ovarian aging and female infertility. However, the production and identification of OSCs are difficult for investigators. Rare amount of these cells in the ovary results in the failure of the acquisition of OSCs. Furthermore, the oocyte formation by OSCs in vivo was usually confirmed using tissue sections by immunofluorescence or immunohistochemistry in previous studies. STO or MEF feeder cells are derived from mouse, not human. In our study, we modified the protocol. The cells were digested from ovaries and cultured for 2-3 days and then were purified by magnetic-activated cell sorting (MACS). The ovaries and fetus of mice injected with EGFP-positive OSCs were prepared and put on the slides to directly visualize oocyte and progeny formation under microscope. Additionally, the human umbilical cord mesenchymal stem cells (hUC-MSCs) were also used as feeder cells to support the proliferation of OSCs. The results showed that all the modified procedures can significantly improve and facilitate the generation and characterization of OSCs, and hUC-MSCs as feeder will be useful for isolation and proliferation of human OSCs avoiding contamination from mouse.

## 1. Introduction

Ovarian aging is characteristic of progressive decline of follicle reservoir, and thus women suffer aging-related health problem and psychological stress. Since 2004, the research of stem cells related with female germ cell commitment emerged, gradually increased, and became a hot spot [1–8]. Female germline stem cells (FGSCs) or oogonial stem cells (OSCs), first reported by Wu group and subsequently by Tilly group [9, 10], demonstrated the existence of a population of germline stem cells in postmammalian ovaries [11]. However, after the onset of isolation and identification of FGSCs/OSCs, the controversy against these observations continues to exist [12–15]. Perhaps this is firstly because no enough comprehensive evidence, especially regenerated oocytes or follicles in vivo from FGSCs/OSCs, was obtained to testify prior observations and challenge traditional paradigm and secondly because generation and characterization of FGSCs/OSCs-related complication hampered the new researchers into this field. For instance, only rare cells were acquired in the process of two-step enzymatic digestion of ovaries from mice, resulting in even minimal cells after magnetic-activated cell sorting (MACS) or fluorescence-activated cell sorting (FACS), which means that it is extremely hard to successfully establish oogonial stem cell lines. In addition, it is comparably difficult for newcomers to perform the experiments on the observation of differentiation into oocytes or progenies. Thus, we attempted to make some modification to facilitate these experiments [9, 10, 16, 17]. So the aim of our study is to facilitate the derivation and identification of OSCs, overcoming the difficulties on the way to obtain the OSC lines and to attract more researchers into the field. Only if more researchers work in this field and publish more comprehensive studies about OSCs, we can determine the true nature of the OSCs to conclude the debate. Initially, we performed the MACS for cell suspension of ovarian tissue 2-3 days after culture of total population of dispersed cells from the digested ovaries; thus, there were more cells and more viable cells for sorting based on antibodies. In addition, 2-3-day culture after digestion can avoid further damage in the process of MACS and restore the viability of cells to some extent. Secondly, identifying the differentiation capacity of OSCs through immunofluorescence or immunohistology on consecutive sections greatly decreases the possibility and increases the difficulty to find the positive oocytes or follicles originating from EGFP-expressing OSCs. Therefore, we developed a novel method to directly visualize the fluorescence from EGFP-expressing oocytes or follicles under microscope. Briefly, the ovaries injected with EGFP-expressing OSCs were dissected; then, these ovaries were mechanically or enzymatically dispersed to release oocytes or follicles which were harvested together with remaining tissues to be visualized on the slides with a cover glass under fluorescence microscopy. Next, we found that the fetus at E12.0 can be visualized under fluorescence microscopy to verify if EGFP-positive mice are generated. This helps the investigators to obtain the outcomes of differentiation as fast as possible and does not need any expensive instruments like live imaging system. Finally, the human umbilical cord mesenchymal stem cells (hUC-MSCs) were employed to support the growth of OSCs, which aim to establish human OSC lines without any contamination from mouse. In brief, using these modifications, the isolation and identification can be easily finished, and the improvement will facilitate and prompt future researches on the oogonial stem cells.

## 2. Materials and Methods

### 2.1. Animals

Six-week-old C57BL/6 mice used in this study were purchased from the Center of Medical Experimental Animals of Hubei Province (Wuhan, China) and the Center of Experimental Animals of Chinese Academy of Medical Science (Beijing, China). All procedures involving animals were approved by the Animal Care and Use Committee of Tongji Medical College and were conducted in accordance with the National Research Council Guide for Care and Use of Laboratory Animals.

### 2.2. Isolation and Culture of OSCs

OSCs were isolated from 6-week-old mice using the methods described previously [9, 10, 16, 17]. Briefly, the ovaries from female mice were dissected and minced into slurry in the collagenase/Dnase I solution (Worthington, USA) and then incubated at 37°C for 20 minutes which was repeated once or followed by trypsin treatment for 5–10 min and finally the trypsin was neutralized by 10% fetal bovine serum (FBS). After centrifugation of suspension and removal of supernatant, the pellet was placed onto 6-well plate without STO feeder layer. Two or three days later, the cells were trypsinized and purified by MACS using Fragilis antibody and goat anti-rabbit IgG microbeads [18]. The sorted cells were cultured onto feeder cells with the medium which consisted of minimum essential medium *α* medium (MEM-*α*) (32561-102, Invitrogen), 10% FBS (06902, Stemcell), 1 mM sodium pyruvate (P2256-25, Sigma), 1 mM nonessential amino acids (11140-050, Gibco), 0.1 mM *β*-mercaptoethanol (ES-007-E, Millipore), 1000 units/mL LIF (ESG1106, Millipore), 1 ng/mL bFGF (13256-029, Gibco), 10 ng/mL EGF (PHG0311L, Gibco), 20 ng/mL human GDNF (212-GD-010, R&D), 1×-concentrated N2-supplement (AR009, R&D), and 1×-concentrated penicillin-streptomycin. Subculture of oogonial stem cells (OSCs) was performed according to reports published previously.

### 2.3. Culture and Preparation of STO Cell Line and hUC-MSCs

The OSCs were plated onto mitotically inactivated STO cell feeders from ATCC. STO cells were cultured in Dulbecco's modified Eagle's medium (DMEM) with high glucose (Life Technologies), supplemented with 1 mM nonessential amino acids (11140-050, Gibco), 2 mM glutamine, 30 mg/L penicillin, 75 mg/L streptomycin, and 10% FBS (Invitrogen), which was described in previous reports. To prepare STO cell feeder, the STO cells were first treated with mitomycin C (10 *μ*g/mL, Sigma) for 2-3 hours and then washed with PBS and plated on 24-well plate.

Human umbilical cord mesenchymal stem cells (hUC-MSCs) were donated from Hubei Key Laboratory of Embryonic Stem Cell Research in Taihe hospital, and the medium preparation and cell culture were performed as described by them [19]. To prepare hUC-MSCs cell feeder, like STO cells, the hUC-MSCs of passages 3–5 were treated with mitomycin (10 *μ*g/mL, Sigma) for 3 hours, washed, and plated on 24-well plate.

### 2.4. Immunofluorescence

OSCs were fixed with 4% paraformaldehyde for 15 min at room temperature and then incubated in blocking solution (10% normal goat serum in PBS) for 1 h at room temperature. Following the incubation at 37°C for 1 h with primary antibodies  (rabbit polyclonal anti-MVH (1 : 200 dilution, ab13840, Abcam), rabbit polyclonal anti-Fragilis (1 : 500 dilution, ab15592, Abcam)), OSCs were incubated with FITC conjugated secondary antibody (goat anti-rabbit IgG, 1 : 1000 dilution) and then were stained by DAPI for 15 min.

### 2.5. Reverse Transcription-Polymerase Chain Reaction

Total RNA was extracted with RNAiso reagent (Takara, China) according to the manufacturer's instructions. Approximately 2 *μ*g of RNA was treated by Dnase I to remove trace amounts of DNA contamination; then, the RNA was used to synthesize cDNA using transcriptor reverse transcriptase (transcriptor cDNA first strand synthesis kit, Roche) following the manufacturer's manual. Finally, the cDNA was performed for PCR amplification and the primers are listed in Table 1 with reference to other reports [9, 10].

### 2.6. Karyotype Analysis of OSCs

After 3 days of OSC passage, the cells were treated with OSC medium supplemented with 80 ng/mL colchicine for 3 h and were then hypotonically treated with 40 mM KCl for 30 min. Following the fixation in methanol-acetic acid (3 : 1) for 1 h, the slides were stained with Giemsa buffer and observed under the microscope.

### 2.7. Self-Inactivation of Lentivectors in OSCs

To observe whether transduced OSCs were unable to produce infectious lentiviral particles, the infected OSCs with EGFP expression were seeded on 6-well plates and cultured to confluency without changing the medium. The supernatant was then collected and filtered through a 0.45 *μ*m pore-sized polyethersulfone membrane, and 1 or 2 mL was incubated with wild-type OSCs without EGFP expression. Then, the EGFP expression of wild-type cells was observed.

### 2.8. Alkaline Phosphatase Staining

Alkaline phosphatase activity was assayed by AP detection kit (1101-050, SiDanSai, Shanghai, China) according to the manufacturer's instructions. Briefly, the cells cultured on the plates were fixed with 4% paraformaldehyde for 1-2 minutes and washed by PBS twice and then incubated by TBST solution. Finally, the AP staining solution was prepared with solutions A, B, and C according to instructions and then was added to the cells for 15 minutes. The cells were then observed under microscope.

### 2.9. OSCs Infection with Lentivirus Vector and Transplantation into Recipient Mice

The lentivirus vector expressing EGFP and its packaged virus particles were purchased from Genechem company (Shanghai, China). The established OSCs were infected according to the company's manual. At least 1 week after infection, the OSCs were trypsinized into cell suspension and about 1 × 10^4^ cells were injected into each ovary of the recipient mice using Nanofil syringe (World Precision Instruments, USA) according to the protocol described previously [9, 16].

### 2.10. Southern Blotting

DNA probe for southern blotting was synthesized by PCR amplification from plasmid DNA carrying EGFP gene as template using the specific primers: 5′-ATGGTGAGCAAGGGCGAGG-3′ and 5′-CGTCCTCGATGTTGTGG-3′. The 523 bp amplification products were electrophoresed and purified. Digoxigenin labeling was done by using the DIG high prime DNA labeling and detection starter kit I (Roche). Genomic DNA was extracted from the tails of the progenies digested with PstI and the digested DNA samples of 25–30 *μ*g were electrophoresed in 0.8% agarose gels. Plasmid DNA was used as positive control. The separated DNA fragments were transferred to 0.45 *μ*m nylon membranes and fixed by UV cross-linking; then hybridization and stringency washes were carried out. Finally, the detection was performed using anti-Anti-Digoxigenin Alkaline phosphatase (AP) and its substrate with the above DIG detection kit (Roche) following the manufacturer's manual.

## 3. Results

### 3.1. Isolation and Long-Term Culture of OSCs

**Figure 1:**
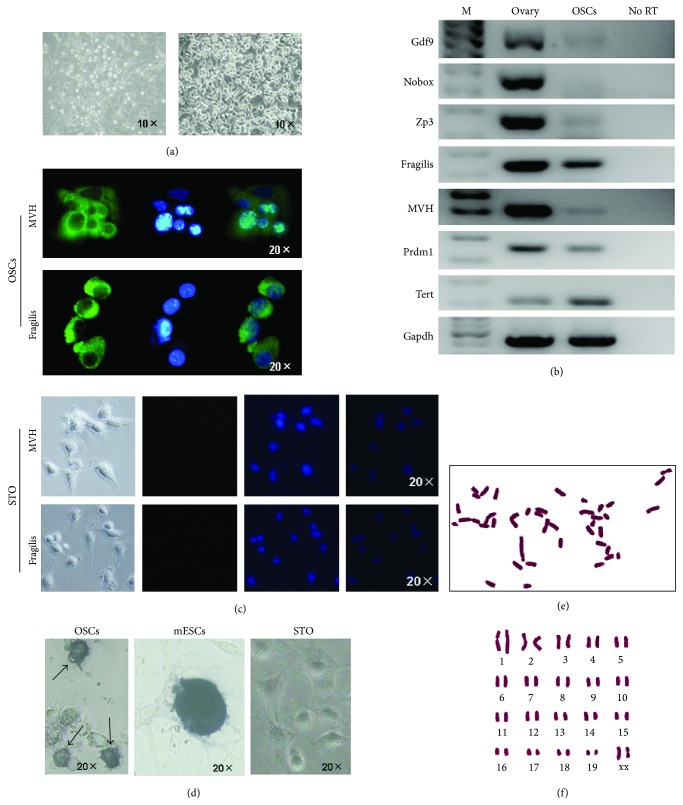
Morphology and characteristics of the established OSCs. (a) Overview of the OSCs immediately after MACS (left) and the established OSCs (right) which formed the typical structure of cell clusters. (b) Reverse transcriptional PCR analysis for expression profile of OSCs and ovarian tissues as the positive control. There were two set of genes: one for oocytes including Gdf9, Nobox, and Zp3 and another one for germ cells including Fragilis, MVH, Prdm1, and Tert, displaying that OSCs are characteristic of germ cells. No RT, PCR of RNA sample without reverse transcription. (c) Immunofluorescence for MVH and Fragilis in established OSCs and STO as negative control. Green, MVH, and Fragilis immunofluorescence; Blue, DAPI. At the bottom are the images (bright field, Green, DAPI, and merge) of STO from left to right. (d) Alkaline phosphatase staining for established OSCs, mESCs, and STO showed that OSCs (arrows) were positive, whereas mESCs were strongly positive. mESCs, mouse embryonic stem cells. (e-f) Cytogenetic analysis for established OSCs showed normal karyotype.

According to improved MACS method by Zou et al., we employed Fragilis as the marker for selection of OSCs [18]. If the dissected ovaries are enzymatically treated and MACS is promptly performed, the purification will likely fail considering that the harvested cells from digested ovaries are in considerably small amount and suffer further damage of their viability due to the two-step preparation including digestion and MACS. So, we cultured the digested cells from 6 ovaries for 2-3 days during which the total number of cells increased to 0.5–1 × 10^5^; then, these cells were used for MACS by antibody of Fragilis and finally 2–5 × 10^4^ cells (about 5%) flushed “positive cells” (including contaminated false-positive cells) were obtained from the total cells. Then, these rare cells were cultured on the STO feeders for about 5–7 days in 24-well plates, and until the cells grew to confluence, they were prepared into cell suspension by trypsin and passaged onto a new 24-well plate with STO feeders. Usually after in vitro culture and proliferation for about 1 month, these purified putative OSCs can be established. The morphology of our established OSCs was the same as previous reports, which represented as ovoid and clustered cells with a large ratio of nuclear plasma (Figure 1(a)). After the OSCs were established in vitro for over 1 month, these cells were cultured in the absence of feeder cells, just like the report by Tilly et al. [10, 16].

### 3.2. Identification of OSCs for Gene Expression, Immunofluorescence, AP Staining, and Karyotyping

After the OSCs were established, these cells were subsequently identified for gene expression profile which displayed the cells having the characteristics of female germ cells, not oocytes (Figure 1(b)). The specific genes for oocytes including Gdf9, Nobox, and Zp3 showed that Gdf9 and Zp3 were weak positive to OSCs, which suggested that during the culture some OSCs have differentiated. The genes for germ cells including Fragilis, MVH, Prdm1, and Tert were positive to OSCs. To extend mRNAs analyses of MVH and Fragilis, which are classic primitive germline markers, proteins coded by both genes were detected and found to exhibit a pattern of membrane or plasma subcellular localization (Figure 1(c)), which agrees with previous reports. These OSCs showed positive staining for Alkaline phosphatase (AP) as compared to strong staining of mouse embryonic stem cells (mESCs) (Figure 1(d)). Finally, we performed karyotyping of the OSCs and the results displayed the normal karyotype in about 62% (Figures 1(e) and 1(f)).

### 3.3. Differentiation of OSCs into Oocytes, Follicles, and Progeny Formation

**Figure 2:**
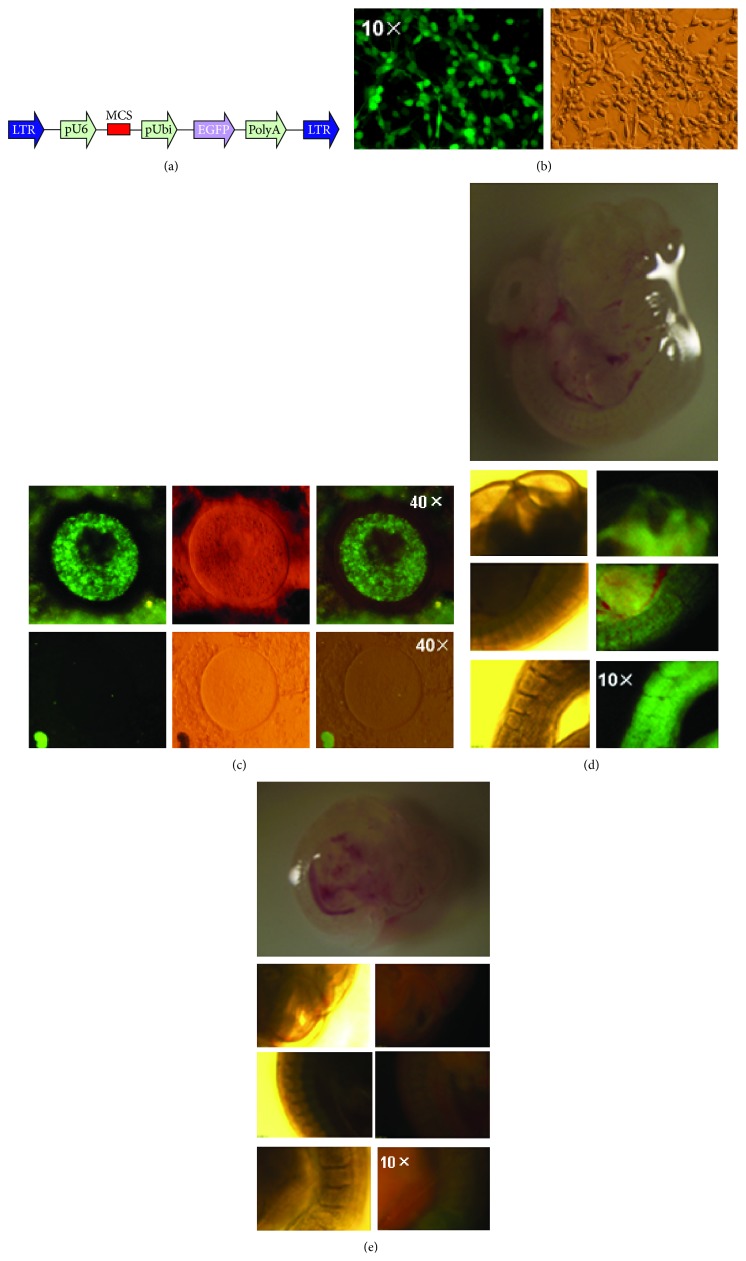
Transfection of OSCs and transplantation into ovaries of recipient mice. (a) Schematic diagram for the lentivirus vector with EGFP. LTR, long terminal repeat; pUbi, ubiquitin promoter. (b) Immunofluorescence-based analysis (right) and bright field view (left) of OSCs transfected with EGFP vector. (c) The ovaries from the recipient were dissected 12 days after injection with EGFP-expressing OSCs and the oocytes within these ovaries were visualized under fluorescence microscope. EGFP-positive oocyte (above) displayed fluorescence and the negative one (below) showed no fluorescence. (d, e) The fetuses from the mated recipients were obtained and observed under fluorescence microscope under the same exposure time. (d) On the top is the overview of the EGFP-positive fetus under stereoscope and at the bottom are the images from the head, body, and tail section showing fluorescence. (e) On the top is the EGFP-negative fetus under stereoscope and at the bottom are the images showing no signal in three sections under fluorescence microscope.

**Figure 3:**
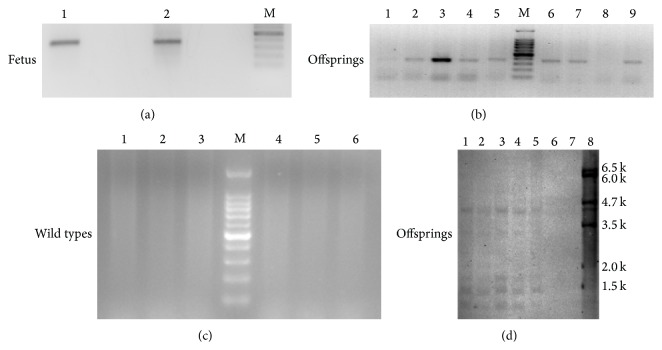
PCR for detection of EGFP gene integrated into the genomes of fetus and offspring. (a–c) Examples of PCR analysis of fetus, offspring and the wild types for detection of EGFP integrated into recipient genomes. There were positive outcomes of 366 bp PCR product in both fetus (a) and offspring (b), but no positive band was found in the wild types (c) which served as the negative control. (d) Southern blot analysis of tail DNA from six offspring, produced from the same recipient mouse and one wild type using a 523-bp PCR product from the control plasmid with primers for EGFP gene as a probe, showing five positive and one negative. Genomic DNA was digested with PstI. Size markers are indicated in the right of southern blot. Lanes 1–6, transgenic mice; lane 7, WT mouse; and lane 8, the control plasmid.

To confirm the oogenic capacity of these putative OSCs, they were stably transfected with a lentivirus EGFP-expressing vector (Figure 2(a)). Due to the concern about the remaining lentivirus particles in the OSCs which can contaminate the endogenous oocyte or follicle population in the ovary, we cultured these EGFP-OSCs and passaged them for at least 1 week to avoid the risk of contamination after successful transfection (most of cells expressed EGFP (Figure 2(b)), hereafter called EGFP-OSCs). At the same time, we conducted self-inactivation of EGFP-OSCs to verify whether these cells can still generate lentivirus particles and the results showed that no EGFP signals were detected in wild-type OSCs suggesting EGFP-OSCs did not generate virus particles. In addition, although most of the OSCs expressed EGFP after transfection, there was still a small amount of OSCs without EGFP expression. Subsequently, approximately 1 × 10^4^ infected EGFP-OSCs were injected into the ovaries of recipient female mice pretreated with cyclophosphamide and busulphan or into the ovaries of wild-type mice. We collected ovaries for retrieval of oocytes and follicles at least 12 days after injection to detect the presence of EGFP-positive oocytes or follicles. These ovaries were slightly dissected and dispersed by needle and then put on the slides with coverslip and were directly visualized under fluorescence microscope. We successfully found some EGFP-expressing oocytes in the ovaries (Figure 2(c)). To our surprise, these EGFP-expressing oocytes were similar with primitive immature oocytes and mature oocytes or follicles were hardly detected by fluorescence. This is likely because EGFP expression was silent in the process of oocyte development in spite of the integration of EGFP gene into genomes of oocytes from OSCs and also because of the very weak signal in matured oocytes due to the pUbi promoter. Subsequently, the recipient female mice were mated with wild-type male mice, and at E12.0 fetuses were collected and placed onto the slides to observe the fluorescence under microscope. We easily found EGFP-positive as well as EGFP-negative samples mainly because fetuses at this stage were thoroughly transparent (Figures 2(d) and 2(e)); so we only need general microscope to distinguish the fetus integrated with EGFP. Finally, the progenies were obtained from the mated recipients which had a rate of 6–9 offspring per pregnant mouse. The genome DNA from the fetus and offspring, together with wild-type mice as negative control was extracted and PCR was performed to screen whether the exogenous EGFP gene was integrated into genome. The results showed that both the samples from fetus and offspring were EGFP-positive; however, the controls were all negative (Figures 3(a)–3(c)). Subsequently, the PCR products from the positive samples were extracted and sequenced, confirming the successful EGFP integration (data not shown). The southern blot analysis was performed to further confirm the integration of EGFP and showed five positive and one negative sample (Figure 3(d)). As shown in the southern blot, lanes 2 and 5 displayed fewer number of integration sites than lanes 1, 3, and 4, which suggested there were two types of transgenic structure in F1 offspring. To sum up, the results suggested that our established OSCs possess the capacity of differentiation into oocytes, ultimately resulting in the generation of progenies.

### 3.4. Comparison between STO and hUC-MSCs as Feeder Cells

**Figure 4:**
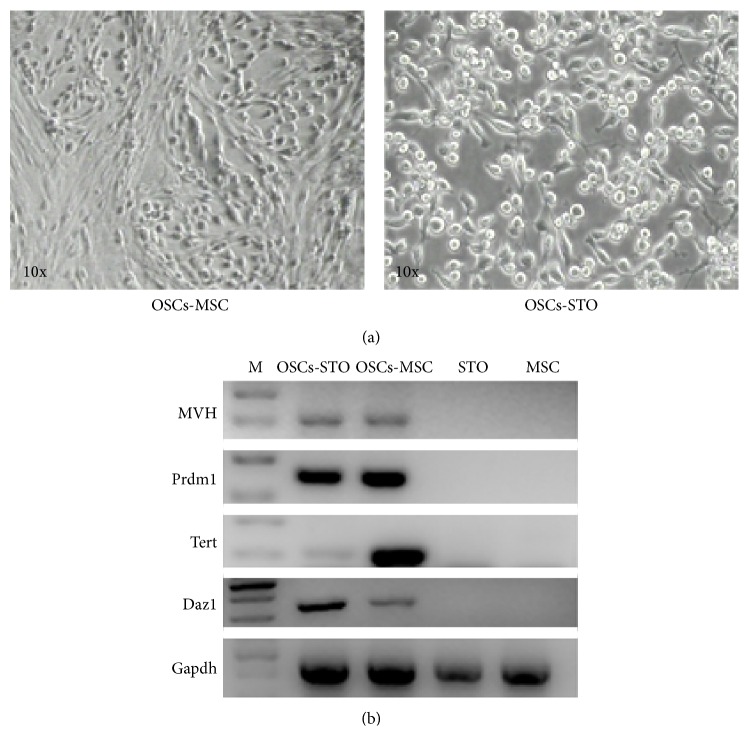
Comparison of STO and hUC-MSCs as feeder cells. (a) Morphological overview showed that OSCs cultured on the hUC-MSCs represented nest-like colonies (left), as compared to the typical cluster-like structures on STO (right). (b) RT-PCR analysis for gene expression profiles of OSCs cultured on STO and hUC-MSCs. The results displayed that, compared to STO feeder, OSCs on hUC-MSCs still retained the germ cell markers. STO and MSC, the negative controls for RT-PCR.

To evaluate whether hUC-MSCs can serve as feeder cells, established OSCs were placed on respective STO and hUC-MSCs. After they proliferated at confluence at passage 2, they were harvested and RNA was extracted for reversed transcriptional PCR (RT-PCR) to examine their expression profiles. OSCs cultured on hUC-MSCs showed the nest-like colony morphology that was distinct from that cultured on STO (Figure 4(a)). However, OSCs on hUC-MSCs still retained germline expression pattern similar to OSCs on STO (Figure 4(b)), which showed that hUC-MSCs could be used as feeder layer for OSCs, especially for human OSCs, so that we can obtain human OSCs free of any mouse cellular contamination in the future.

## 4. Discussion

The presence and validation of FGSCs or OSCs are of significance for reproductive biology; therefore, some researchers paid attention to whether or not oogenesis occurs from these stem cells in vivo in the adult mammalian ovary [12–15]. In return, supporters of OSCs also criticized their investigations by showing that various adult body organs possess two types of stem cells including active and also dormant stem cells [16, 20–23], which may suggest in vivo OSCs can represent the dormant state as well, resulting in no oogenesis. In addition, other types of germ stem cell in adult ovary have been reported which suggests the existence of niche for germ stem cell in ovary [24–29]. Factually, identification and isolation of FGSCs/OSCs clearly demonstrated their existence although the in vivo counterpart of OSCs showed no direct evidence for active differentiation into newborn follicles after birth. Practically, we could call this state of in vivo OSCs “silence” or “mitotically active state” rather than deny the existence of these cells [11]. Also, in vitro cultured OSCs have been a great tool to researchers; for instance, the OSCs were used for attempts in in vitro differentiation into oocytes [30], in generation of transgenic animals [31, 32], and in cell therapy [33]. Therefore, the isolation and related researches of OSCs should be prompted which will ultimately settle the controversy on this issue.

The reason for selection of 2-3 days after culture of dispersed ovarian tissues is that this period of time can enable the cells to not only restore its viability from the damage of prior treatment but also reach the optimum amount of cells for MACS. In addition, within 2-3 days, the cells can also retain the initial state to the maximal extent as in vivo. Alternatively, selective adhesion method can be chosen to remove large amount of granulosa cells and stromal cells, and this method can enable dispersed cells to proliferate for even longer time because the period of selective adhesion is usually about 7 days, and ultimately more putative OSCs can be obtained after MACS sorting.

The capacity of differentiation into oocytes and progeny is most relevant to the OSCs. However, previous studies observed and evaluated the oocytes and follicles formation all through consecutive sections and subsequent staining such as immunofluorescence. Although these methods effectively helped the investigators to reach their goals, yet it is not a unique choice compared with the method employed in our studies. It is more optimal if the EGFP fluorescence signal can be directly visualized under general fluorescence microscope. In our experiments, we indeed found some EGFP-expression oocytes; however, only few oocytes displayed the fluorescence signal. It is known that pUbi promoter drives weak expression of the downstream gene and that the expression of exogenous gene such as EGFP is complicated by the integrated location in the genome. If the CMV or other optimal promoters are to be used in this study, the EGFP expression can be easily detected and perhaps be observed in the majority of follicles in ovaries. Since the outcome from this method has some limitation, we should optimize the method to easily detect the EGFP-positive oocytes or follicles. In addition, due to the concern about the possible contamination from the virus particles generated from the transduced OSCs, we performed the test to verify whether these cells produced particles [34] and the results showed that the EGFP-positive oocytes detected cannot be false-positive because no fluorescence was observed by the wild-type cells as control. Finally, considering that we did not purify the transduced OSCs by FACS, the EGFP-OSCs invariantly included some untransduced OSCs whose oocytes could not express EGFP.

Likewise, the fetuses at E12.0 were selected to examine if EGFP-expressing progeny was produced because they are transparent and can be easily visualized under general fluorescence microscope. Indeed, to some extent, visualization of EGFP was more easy and more sensitive under fluorescence microscope than live imaging system. Moreover, this method is more convenient and can provide investigators with more confidence to perform their identification tests. However, the limitation from the weak promoter influenced the observation; thus, based on our experience, the fetus for fluorescence screening should be earlier than E12.0 because the size of fetus more than E12.0 usually becomes larger and therefore hardly transparent. In addition, EGFP expression is much complicated in transgenic animals resulting in no EGFP expression in some organs of offspring; however, all the organs of the fetus can be easily screened and thus do not suffer due to the limitation. Additionally, the fetuses were subsequently used for PCR screening. The results were consistent with the observation under fluorescence microscope. We also used the genome DNA extracted from the positive fetus for southern blot analysis in another study and found that it is as feasible as tail DNA from the offspring. In contrast to the PCR, southern blot analysis can supply detailed information about the genetic structure of the transgenic alteration, for example, transgene copy number and the number of integration sites within the genome. As shown in Figure 3(d), we found that multiple insertions occurred in offspring which suggested the successful integration of EGFP gene.

In summary, we demonstrated the modifications on the MACS selection and identification of mouse OSCs, including presort culturing for 2-3 days, and the direct visualization of EGFP-positive oocytes and fetus. In addition, hUC-MSCs as the feeder layer will be useful to the clinical application for human OSCs as well. Although there are restrictions in our study, the outcome of this study may facilitate the research on the OSCs and attract more researchers into this field to make novel investigations to settle the remaining debate about OSCs.

## Figures and Tables

**Table 1:** Details regarding PCR primers used in RT-PCR for mouse ovary and OSCs.

Gene	Accession number	Product size (bp)	Primer sequence (5′-3′)
Gdf9	NM_008110	709	F: TGCCTCCTTCCCTCATCTTG R: CACTTCCCCCGCTCACACAG

Nobox	NM_130869	379	F: CCCTTCAGTCACAGTTTCCGT R: GTCTCTACTCTAGTGCCTTCG

Zp3	NM_011776	183	F: CCGAGCTGTGCAATTCCCAGA R: AACCCTCTGAGCCAAGGGTGA

Fragilis	NM_012013	151	F: GTTATCACCATTGTTAGTGTCATC R: AATGAGTGTTACACCTGCGTG

MVH	NM_010029	216	F: ACCCAGTTTGGTCATTCAGTTCG R: TTGTTCCTTTGATGGCATTCCTG

Prdm1	NM_007548	149	F: ACAGAATGGCAAGATCAAGTATGA R: GGTGGGCGAGCTGAGTAAAA

Tert	NM_009354	120	F: GCTTCCCTTTGACCAGCGTGTTA R: GCCTTTAGTGTCATTCCTGGATTCTT

Dazl	NM_010021	358	F: GTTAGGATGGATGAAACCGAAAT R: ATGCCTGAACATACTGAGTGATA

Gapdh	NM_008084	458	F: GTCCCGTAGACAAAATGGTGA R: TGCATTGCTGACAATCTTGAG
